# Rural Epidemiology of HIV Positive Tribal Patients from Chhattisgarh in India

**DOI:** 10.4103/0974-777X.59249

**Published:** 2010

**Authors:** Harminder Singh, Naveen Dulhani, Nel Kumar Bithika, Pawan Tiwari, VKS Chauhan, Prabhakar Singh

**Affiliations:** *Department of Pharmacology, Medicine & PSM, Govt Medical College, Jagdalpur, Chhattisgarh, India*; 1 Pharmacology, SS Medical College, Rewa, MP, India

**Keywords:** Human immune deficiency virus, People living with HIV/AIDS, National aids control organization, Pyrexia of unknown origin, Highly active antiretroviral therapy

## Abstract

**Objective:**

The primary objective was to study the epidemiology of Human Immunodeficiency Virus (HIV) positive tribal patients, and the secondary objective was to study the associated comorbidities in a tertiary care hospital in the tribal (Bastar) region of Chhattisgarh, India, between December 2006 and November 2008, and their relation to CD4 counts.

**Materials and Methods:**

In this study 90 tribal HIV positive subjects were enrolled. Information on demographics, that is, weight, height, age, educational status, sex, clinical finding, and laboratory parameters (CD4 counts) were noted.

**Results:**

Among 90 HIV patients, 54 (60%) were males and 36 (40%) were females. Among these, most patients, 37 (41.1%), were in the age group of 30 to 39 years. Among these patients, 79.56% belonged to the lower socioeconomic status, whereas, only 1.45% were from a high socioeconomic status. The largest group was made up of drivers (32.2%), with the second largest group being housewives (27.7%) and laborers (17.7%), respectively. A majority of the patients had a low education, 35.5% were educated only up to the fifth standard and 31.8% up to high school, while 18.8% were illiterate. The predominant mode of transmission was heterosexual contact (78.8%), only one patient (1.1%) was infected through transfusion of infected blood, five (5.5%) patients acquired infection via vertical (mother to child) transmission, and in 13 patients the transmission history was not clear.

**Conclusion:**

There was a high frequency of behavioral risk factors, together with unawareness, and very little health infrastructure, thus creating an impending risk for the rapid spread of HIV/AIDS (acquired immunodeficiency syndrome).

## INTRODUCTION

A total of 39.5 million (34.1 million-47.1 million estimated) people were living with HIV in 2006.[1] This includes the estimated 4.3 million (3.6 million-6.6 million) adults and children who were newly infected with HIV in 2006. Newer, more accurate estimates indicate that approximately 2.5 million (2 million-3.1 million) people in India were living with HIV in 2006, and the adult national HIV prevalence was 0.36%, although the proportion of people living with HIV is lower than previously estimated.[2] The highest number of PLHA is in Andhra Pradesh and Maharashtra, while Manipur and Nagaland have the highest prevalence, due to their small population size. According to NACO 2006, HIV prevalence in Chhattisgarh was 0.17%; it was maximum (1.67%) in Manipur and minimum (0.03%) in Himachal Pradesh.[3] In Chhattisgarh, the Durg district has the maximum HIV burden and Bastar the second highest. These are categorized as categories A and C, respectively.[4] The epidemiological features depend on the social and cultural practices of those people, which may again vary from region to region, and the clinical features and opportunistic infections of HIV infection may depend on the organisms and parasites endemic in that country.[5,6]

There are increased rates of hepatic, cardiovascular, metabolic, and/or endocrine, renal, neurological, and pulmonary events also among HIV-infected patients.[7]

We have the second largest pool of HIV-infected patients, but very little is known about the comorbidity conditions for which patients are admitted to hospital.[8] In the present study we have described the tribal HIV problem epidemiologically, clinically, and with laboratorial evaluation.

## MATERIALS AND METHODS

This is an observational study of a cohort of patients receiving longitudinal HIV primary care in the Department of Medicine, Government Medical College and the associated Maharani Hospital, in Jagdalpur (Chhattisgarh).

The sample consisted of 137 consecutive HIV-positive patients who visited the tertiary hospital for medical care between December 2006 and November 2008. Those with a non-tribal background, denied written consent, migrated to another part of the state or were lost to follow up, were excluded from the study (n = 47). Finally 90 (n = 90) patients were eligible for inclusion in the study were those tribal HIV patients who were regular with their follow-up and were evaluated epidemiologically, clinically, and laboratorically.

Sampling: Being a prospective and observation study we included all HIV-positive patients who came to seek medical care in our hospital.

Data collection: All enrolled tribal HIV positive patients (n = 90) were seen by the treating physician and the relevant data was collected, which included information on demographics, for example, age, sex, height, weight, socioeconomic status (SES), educational status, laboratory parameters (CD4 + counts), and full clinical evaluation including dermatological findings were noted. Various samples, for example, sputum, oral swab, blood, stools, urine, cerebrospinal fluid (CSF), and lymph node aspirate were collected as per symptoms and clinical presentations, under universal aseptic precautions, in suitable sterile containers.

Data was processed and analyzed with SPSS version 12.0. The demographic and opportunistic infections data were expressed in percentages. The prevalence of common opportunistic infections was stratified by the CD4 cell count at presentation, and the chi-squared statistic was used to detect differences between the strata. *P* < 0.05 was considered statistically significant.

Ethical consideration: The study was initiated after taking the approval of the institutional Human Ethic's Committee and written consent from the patients. As a protocol, identities of the enrolled subjects were kept confidential, and names and personal identities were not disclosed in any concerned document.

## RESULTS

### Demographic profile

Results of this study showed that among 90 HIV patients, 54 (60%) were males and 36 (40%) were females. Among these, most patients, 37 (41.1%), were in the 30 to 39 age group. Among these patients, 79.56% belonged to the lower socioeconomic status, whereas, only 1.45% came from the high socioeconomic status. Among our subjects, the largest group comprised of drivers (32.2%), with the second largest group being housewives (27.7%) and laborers (17.7%), respectively. A majority of the patients had low education; 35.5% were educated only up to fifth standard and 31.8% up to high school, while 18.8% were illiterate [Table 1].

**Table 1:** Demographic parameters of HIV-infected patients in the study

	No. of cases (%)
Age in years	
Below 19	05 (5.5)
20-29	29 (32.2)
30-39	37 (41.1)
40-49	13 (14.4)
50-59	05 (5.5)
>60	01 (1.1)
Grand total	90
Sex wise distribution	
Male	54 (60)
Female	36 (40)
Grand total	90
Occupations	
Government servants	8 (8.8)
Housewives/spouses	25 (27.7)
Farmers	5 (5.5)
Business	7 (7.7)
Laborers	16 (17.7)
Drivers	29 (32.2)
Grand total	90
Educational status	
Illiterate	17 (18.8)
Below fifth standard	32 (35.5)
High school	28 (31.8)
Undergraduate	10 (11.1)
Post graduate	3 (3.3)
Total	90

### Mode of transmission

The predominant mode of transmission was heterosexual contact (78.8%), only one patient (1.1%) was infected through transfusion of infected blood, five (5.5%) patients acquired infection via vertical (mother to child) transmission, and in 13 patients the transmission history was not clear. There was no history of intravenous drug abuse or homosexual mode of transmission.

### Unique sexual practices among tribal members

Regarding sexual practices, 30% of the respondents reported either premarital affairs or extramarital affairs [Table 2]. However, such practices were seen more in men than in women. Furthermore, 25.5% of the male participants reported having had sex with a commercial sex worker (CSW) [Table 2].

**Table 2:** Self-reported sexual practices information

Character	Finding in n (%)
Age at first sexual activity	
Male	19 (±3.2)
Female	15 (±2.1)
Premarital or extramarital sexual exposure	
Male	22 (19.8)
Female	09 (10)
Commercial sex worker sex exposure	23 (25.5)

### Associated opportunistic infection

The most common opportunistic infection was tuberculosis seen in 21 patients (23.3%) followed by oropharyngeal candidiasis in 15 (16.6%), onychomycosis in 14 (15.5%), bacterial pneumonia in five (5.5%), herpes simplex in three (3.3%), cryptococal meningitis in two (2.2%), and Herpes zoster in two (2.2%). The most common presenting complaints were pyrexia of unknown origin, seen in 45 patients (50%), followed by diarrhea in 27 (30%), weight loss in 19 (21%), and cough in 12 (13.33%) [Table 3]. We found a significant inverse correlation between opportunistic infection and a decline in CD4 cell count [Table 4].

**Table 3:** Most common presenting symptoms

Symptoms	Number % of total
Pyrexia of unknown origin	45 (50)
Diarrhea	27 (30)
Weight loss	19 (21.1)
Cough	12 (13.33)
With dermatological symptoms	9 (10)
Without any symptoms	25 (27.7)

**Table 4:** Correlation, between CD4 cell count and opportunistic infections

CD4 cell count	Patients with opportunistic infection	Patients without opportunistic infection	Total	Chi-square teat result
More than 350	25	20	45	*P* = 0.0115 Significant
Less than 350	37	8	45	
Total	62	28	90	

## DISCUSSION

Results of the present study showed several facts about HIV in the tribal population of Bastar. A majority (90%) of the infected patients belonged to the lower socioeconomic strata, with income less than Rs. 1500 per month. The predominant mode of transmission was heterosexual contacts (78.8%); with comparison to study by Wig N *et al*., which has reported that the mode of transmission was 44.2% heterosexual, 1.4% intravenous drug use, and 17.4% iatrogenic, respectively.[9] The most vulnerable age group was that between 30 and 39 years, with more males affected than females. A similar finding was shared in the study by Naik *et al*.[10]

We found tuberculosis (TB) to be the most common opportunistic infection (23.3%), other studies also show a similar result.[5,6,11] However, Singh *et al*. showed the Oral candidiasis was the most common (59.00%) opportunistic infection.[12] A report from Chennai in 2006, identified TB as a reason for admission in one-third of the patients, which is similar to the finding in the present study.[13]

As Chhattisgarh has one of the highest prevalence of leprosy in India,[14] one case of lepromatous leprosy coinfection was noted in our study, which is very rarely reported in other Indian studies.[15]

Tribal people in our study have sexual practices that differ from those of mainstream cultures. The early age of marriage, forced migration due to unemployment, naxal problem in southern Chhattisgarh, heavy trucker movement in the iron ore region, and exposure to commercial sex workers — all these factors contribute to the unchecked HIV spread. This point is also shared by other studies on tribal population.[16]

This is the first HIV-related study in southern Chhattisgarh, in which we attempted to assess the risk threshold for the transmission and spread of HIV/AIDS. During our study we found that knowledge and awareness about HIV/AIDS and sexually transmitted diseases (STDs) was very low here. The other finding that was surprising was that in spite of a large pool of HIV patients and their related activities, a national organization like NACO had a very poor presence in this part of the state.

Epidemiology and the comorbidities were the primary objectives and as a secondary outcome we found a statistically significant relation between the CD4 cell count and the incidence of opportunistic infection. Similar findings are shared by other studies.[17,18]

## CONCLUSION

HIV infection is one of the major infectious diseases in this part of India, and being chronic and lifelong in nature; its impact is huge compared to other diseases. People with high-risk behavior and spouses of affected patients need to be educated for primary and secondary prevention. The disease results in the additional burden of coinfection and comorbidities, therefore, early recognition of the disease processes will not only prolong survival, but will also decrease the viral load and transmission.

Prevention is the best strategy for reducing the human and economic toll from HIV/AIDS. To have the largest impact on the HIV epidemic, a comprehensive approach is needed for HIV prevention. Comprehensive HIV prevention is a broad term that incorporates surveillance, research, prevention interventions, and evaluation. More research is needed to better define and understand the HIV/AIDS epidemic in terms of behavioral, laboratory, and medical science, and to work to contain the spread of HIV and AIDS.

There is a high frequency of behavioral risk factors, together with unawareness, and too little health infrastructure, thus creating an impending risk for the rapid spread of HIV/AIDS. It therefore becomes imperative and urgent to address the health concerns revealed in our study in order to implement effectual, appropriate, and culture-receptive intervention programs, so that an impending disaster in this inaccessible community can be averted.
